# Screening the key genes of prognostic value in the microenvironment for head and neck squamous cell carcinoma

**DOI:** 10.1097/MD.0000000000024184

**Published:** 2021-01-29

**Authors:** Di Meng, Tongjun Liu, Feng Ma, Mingguo Wang

**Affiliations:** Department of Stomatology, Central Hospital Affiliated to Shandong First Medical University, Shandong Province, China.

**Keywords:** bioinformatic analysis, gene, head and neck squamous cell carcinoma, survival, tumor microenvironment

## Abstract

Head and neck squamous cell carcinoma (HNSCC) is the sixth common malignancy worldwide. The tumor microenvironment is highly related to tumor initiation, progression, and prognosis. This study aims to screen the tumor microenvironment related key genes of prognostic value for HNSCC.

The gene expression and clinical data for HNSCC were downloaded from the cancer genome atlas (TCGA). The immune/stromal/ESTIMATE scores were downloaded from the website of the MD Anderson Cancer Center. Correlation of patient gender and tumor grade with immune/stromal/ESTIMATE score was tested. Patients were divided into low and high immune/stromal/ESTIMATE score subgroups. Survival analysis was performed to evaluate the prognostic value of the immune/stromal/ESTIMATE score. Tumor microenvironment related differentially expressed genes were determined and applied for functional enrichment analysis and protein-protein interaction network was predicted. The prediction value of the common differentially expressed genes on patient survival was tested.

Four hundred eighty samples with complete clinical, expression data, and immune/stromal/ESTIMATE scores were enrolled for analysis. Immune/stromal/ESTIMATE score was higher in female patients than males. A total of 44 common differentially expressed genes were screened in high and low immune/stromal/ESTIMATE score subgroups. Of the 44 genes, 7 genes (ADGRG7, CSN3, CST8, KRT81, MUC7, MYH6, and SEZ6) were found to be closely related to patient survival. Enrichment analysis showed that the differentially expressed genes mainly enriched in the protein-coupled receptor signaling pathway, extracellular region, G-protein coupled receptor activity, salivary secretion, and regulation of lipolysis in adipocytes. Protein-protein interaction analysis revealed that POSTN and OGN were crucial microenvironments related genes.

Tumor microenvironment related genes ADGRG7, CSN3, CST8, KRT81, MUC7, MYH6, and SEZ6 are valuable predictors for HNSCC patient survival. POSTN and OGN are crucial in modulating the microenvironment and tumor biology for HNSCC.

## 1 Introduction

Head and neck squamous cell carcinoma (HNSCC) accounts for 90% of non-cutaneous head and neck malignancies and ranks the sixth most common cancer globally.^[1,2]^ HNSCC is composed of a heterogeneous group of tumors developing from the mucosa of the nasal and oral cavity, oropharynx, hypopharynx, or larynx.^[3,4]^ Great progress has been seen in recent years in the treatment of HNSCCs, including surgery, radiotherapy, chemotherapy, as well as gene-based therapy and immunotherapy.^[4–6]^ For limited or early-stage disease, surgery, or radiation alone are preferred. And for most patients with locally advanced disease, sequential therapy including platinum-based chemoradiation with or without induction chemotherapy is employed.^[7]^ The current standard of care for locally recurrent disease (without surgery or radiation treatment options) and/or metastatic disease has been platinum-based doublet chemotherapy with cetuximab as the first-line therapy.^[7,8]^ However, the prognosis of HNSCC remains poor, with a 5-year mortality rate of nearly 50%, and a median survival time of about 10 months for metastatic HNSCCs, including patients who received combined therapy of cetuximab and chemotherapy (cisplatin and 5-fluorouracil).^[9]^ Recently, immunotherapy agents targeting programmed cell death pathway such as nivolumab (Opdivo, Bristol-Myers Squibb), prembrolizumab (Keytruda, Merk) were approved by the US Food and Drug Administration for selected cases.^[8]^ Although these agents have introduced a relatively better prognosis in selected cases, the majority of HNSCC patients will still progress.^[8]^ Specific prognostic biomarkers are being evaluated to better determine who will benefit from these agents, including programmed death-ligand 1 expression, tumor mutational burden, and immune gene signatures within both the tumor and the surrounding tissue.^[8]^

Tumor cell-intrinsic genes have been widely studied and accepted as the masters in regulating HNSCC initiation, progression, and evolution.^[10]^ A variety of targeted gene therapy strategies have been proposed for HNSCC and got some improvement for patient survival.^[11,12]^ Recently, the tumor microenvironment has attracted scientists attention for elucidating the cross-talk between tumor cells and their surrounding neighbors and detecting novel targeted therapy.^[3,13]^

The tumor microenvironment is the cellular milieu where the tumor is located and consisted of immune cells, mesenchymal cells, endothelial cells, along with inflammatory mediators and extracellular matrix molecules.^[3,14]^ Immune and stromal cells are the 2 major types of non-tumor components in the tumor microenvironment and have been proposed to be valuable for tumor diagnosis and outcome prediction.^[15,16]^ HNSCC microenvironment is characterized by some unique features, leading to immunosuppression and diminished anticancer immunity.^[3]^ Decreased number of immune cells with antigen-presenting machinery and in cytotoxic ability results in profound immunodeficiency and desmoplastic stromal fibroblasts cells promote tumor invasion and progression via autocrine and paracrine factors.^[3]^ To calculate the fraction of stromal and immune cells in tumor samples, Yoshihara K et al proposed an algorithm named ESTIMATE (Estimation of Stromal and Immune cells in Malignant Tumor tissues using Expression data).^[16]^ With this algorithm, the authors could calculate the immune, stromal, and the overall ESTIMATE score by analyzing the specific gene expression signatures of immune and stromal cells.^[16]^ The ESTIMATE algorithm has been used in prostate cancer,^[17]^ breast cancer,^[18]^ gliomas,^[15]^ and colon cancers.^[19]^ Many tumor microenvironment related key genes have been screened, providing potential therapeutic targets for these tumors. Investigations of immune and stromal related genes in HNSCC have not been reported in details.

In this study, with the database from the Cancer Genome Atlus (TCGA) and the ESTIMATE algorithm derived immune, stromal, and ESTIMATE scores, we screened the microenvironment related key genes associated with patient survivals in HNSCC.

## 2 Materials and methods

### 2.1 Database

RNA expression and clinical data such as gender, age, histological type, survival, and outcome were downloaded from the TCGA data portal (https://portal.gdc.cancer.gov/repository). The immune/stromal/ESTIMATE score of HNSCC was downloaded from the MD Anderson Cancer Center (https://bioinformatics.mdanderson.org/estimate/). The RNA expression data, clinical data, and microenvironment related scores were merged with R (Version 4.0.0, https://www.r-project.org/) for further analysis. This study was approved by the ethics committee of our hospital.

### 2.2 Identification of differentially expressed genes (DEGs)

R (Version 4.0.0, https://www.r-project.org/) package EdgeR, pheatmap, and ggplot2 were used to identify the DEGs in high and low immune/stromal/ESTIMATE scores subgroups. Log of fold of change >2 and adjusted *P* value <.05 were set as the cut-off values to screen the DEGs. The DEGs were plotted as a volcano plot. The common DEGs by immune, stromal, and ESTIMATE scores were extracted and plotted by the R package of VennDiagram.

### 2.3 Enrichment analysis of DEGs

Functional enrichment analysis of the DEGs was performed by the online tool of DAVID (The Database for Annotation, Visualization, and Integrated Discovery, https://david.ncifcrf.gov/). The gene ontology (GO) categories by biological processes (BP), molecular functions (MF), and cellular components (CC) were identified. Similarly, the DAVID database was also used to perform pathway enrichment analysis with reference from the KEGG (Kyoto Encyclopedia of Genes and Genomes) pathways. False discovery rate (FDR) < 0.05 was used as the cut-off value. The enrichment of the DEGs in these pathways was demonstrated as the bubble plots with the package of ggplot2 in R.

### 2.4 Construction of the Protein-Protein-Interaction (PPI) network

The protein-protein interaction network was retrieved from the online database of STRING (https://string-db.org/)^[20]^ and visualized by Cytoscape software (version 3.8.0, https://cytoscape.org/).^[21]^ The connectivity degree of each node of the network was calculated. Molecular Complex Detection (MCODE) plugin was used to find clusters based on the topology to locate densely connected regions. The minimum required interaction score in PPI was set as 0.40, and the largest network for each group was demonstrated and connected nodes were included for analysis.

### 2.5 Statistical analysis

Statistical analysis and figure plotting in this manuscript were performed by R (Version 4.0.0, https://www.r-project.org/). The comparison of the immune score, stromal score, and ESTIMATE score between females and males was tested by Wilcoxon random sum test, respectively. The comparison of the immune score, stromal score, and ESTIMATE score among different tumor grade was conducted by Kruskal–Wallis Rank Sum Test. Kaplan-Meier plots were generated to illustrate the relationship between patients overall survival and gene expression levels of DEGs, and the relationship was tested by the log-rank test. Differences with a *P* value of <.05 was deemed as significant. The main packages used in the manuscript include edgeR, ggplot2, VeenDiagram, DescTools, pheatmap, and survival.

## 3 Results

### 3.1 The immune/stromal/ESTIMATE score is significantly related to patients gender

**Figure 1:**
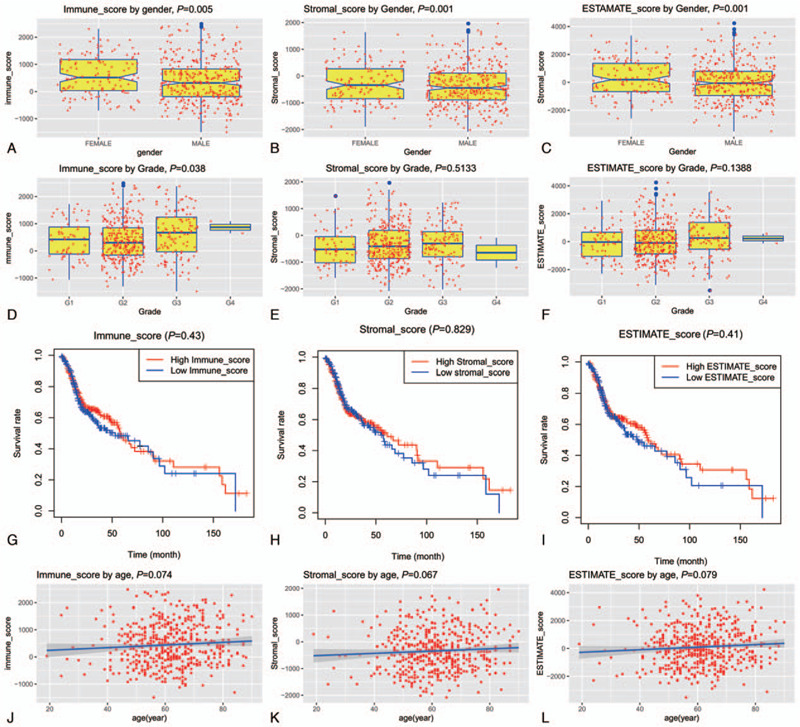
Correlation of Immune/Stromal/ESTIMATE score with clinical features. The Immune/Stromal/ESTIMATE score is significantly higher in female than male patients (A-C). The Immune/Stromal/ESTIMATE is not related to tumor grade (D-E). Although there is a trend that patients with high Immune/Stromal/ESTIMATE score survived better than those with low Immune/Stromal/ESTIMATE, the difference is not significant (F-I). No close correlation exists between patient age and Immune/Stromal/ESTIMATE score (J-L).

At the time of preparing this manuscript, a total of 502 tumor samples and 44 normal samples with gene expression data were available from the TCGA. The clinical data were available for 528 samples. As to immune/stromal/ESTIMATE score, 522 samples were available. After omitting the samples with incomplete data, a total of 480 patients with complete clinical information (gender, grade, follow-up time, and vital status), tumor gene expression data, and immune, stromal, and ESTIMATE scores were enrolled for analysis in this study. The characteristics of the enrolled patients were summarized in Table 1. As Table 1 showed, the mean age of patients in this series was 61.41 ± 11.78 years. Of the 480 patients, 130 (27.1%) were females and 350 (72.9%) were males. The median age in female patients was older than in male patients (65 [58–76] vs 60 [53–67], *P* < .001). As to tumor grade, 61 (12.7%) were grade I, 298 (62.1%) were grade II, 119 (24.8%) were grade III and the rest 2 (0.42%) were grade IV. Based on the ESTIMATE algorithm, the immune score ranged from −1492.71 to 2484.79, the stromal score ranged from −2080.72 to 1957.53, and the ESTIMATE score ranged from −3510.84 to 4227.67. The immune score was significantly higher in female patients than male patients (515.65 vs 316.68, *P* = .005, Fig. 1A). Similarly, the stromal score and the overall ESTIMATE score was also higher in female patients than male patients (-340.41 vs -445.09, *P* = .001 for the stromal score, and 191.14 vs -60.20 for ESTIMATE score, Fig. 1B and C). As to tumor grade, only the immune score was significantly different (*P* = .038, Fig. 1D), but for paired comparison, no differences were found (*P* > .05 for each paired subgroup). Stromal and ESTIMATE scores were not correlated with tumor grade (*P* = .513 and .519 respectively, Fig. 1E and F). To detect the potential correlation of overall survival and immune/stromal/ ESTIMATE score, we classified the patients into high and low immune/stromal/ESTIMATE score subgroups according to their median value (366.685, −400.46, and 19.255 for immune/stromal/ESTIMATE score, respectively), and the survivals between these subgroups were compared. As Figure 1G to I showed, the overall survival was not different between the high and low immune/stromal/ESTIMATE subgroups (*P* = .430, .829 and .410, respectively). Furthermore, patient age was not correlated to immune/stromal/ESTIMATE score (Fig. 1J to Fig. 1L, *P* = .074, .067, and .079, respectively)

**Table 1:** Characteristics of the patients.

characteristics	Patients Number (N = 480)
Gender	
Female	130 (27.1%)
Male	350 (72.9%)
Age (year)	61.41 ± 11.78
Pathology grade	
I	61 (12.7%)
II	298 (62.1%)
III	119 (24.8%)
IV	2 (0.42%)

### 3.2 Identification of DEGs with immune/stromal/ESTIMATE scores

**Figure 2:**
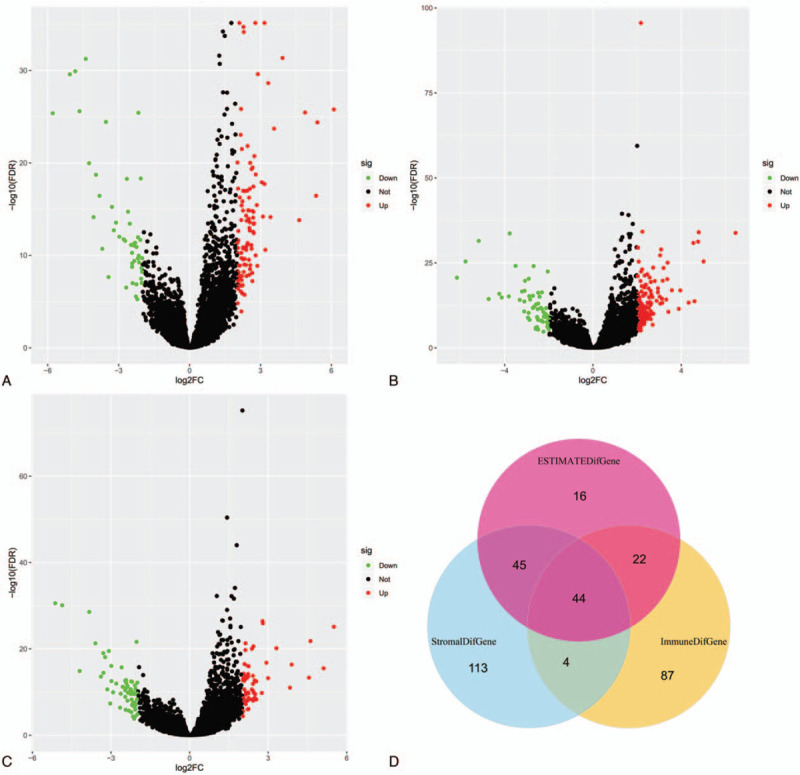
Identification of the differentially expressed genes in high and low Immune/Stromal/ESTIMATE score subgroups. Volcano plots demonstrating the differentially expressed genes in high and low Immune (A), Stromal (B), and ESTIMATE (C) score subgroups. The common differentially expressed genes in high and low Immune/Stromal/ESTIMATE score subgroups (D).

To reveal the correlation of global gene expression profiles with immune/stromal/ESTIMATE scores, we compared the expression profiles between high and low immune/stromal/ESTIMATE subgroups and screened the DEGs. Figure 2 A to C showed the DEGs between these subgroups. One hundred fifty seven differently expressed genes (110 upregulated and 57 downregulated genes) were identified between high and low immune score subgroups (Fig. 2A). Two hundred six differently expressed genes (144 upregulated and 62 downregulated genes) were identified between high and low stromal score subgroups (Fig. 2B). One hundred twenty seven differently expressed genes (64 upregulated and 63 downregulated genes) were identified between high and low stromal score subgroups (Fig. 2C). Among these DEGs in these 3 groups, 44 genes were commonly differently expressed (Fig. 2D). They were GPR146, FABP4, GFY, ADGRG7, MUC7, PSG7, CCDC181, SCGN, NEUROD2, CST8, CCER1, MMP8, UGT2A1, FRMD1, KRT81, DEFB130, NOL4, SEZ6, OR7D4, SMR3B, MS4A12, CGA, KCNH6, CST5, PAH, DMRTB1, HAO1, HIST1H4A, OR5H2, TAC3, FGF3, AQP2, ADCY8, DEFB118, DDC, ITLN1, KCNC2, KRT25, OR2T29, TRHR, MYH6, OR2M4, TSPY3, and CSN3.

### 3.3 Functional analysis of these common DEGs

**Figure 3:**
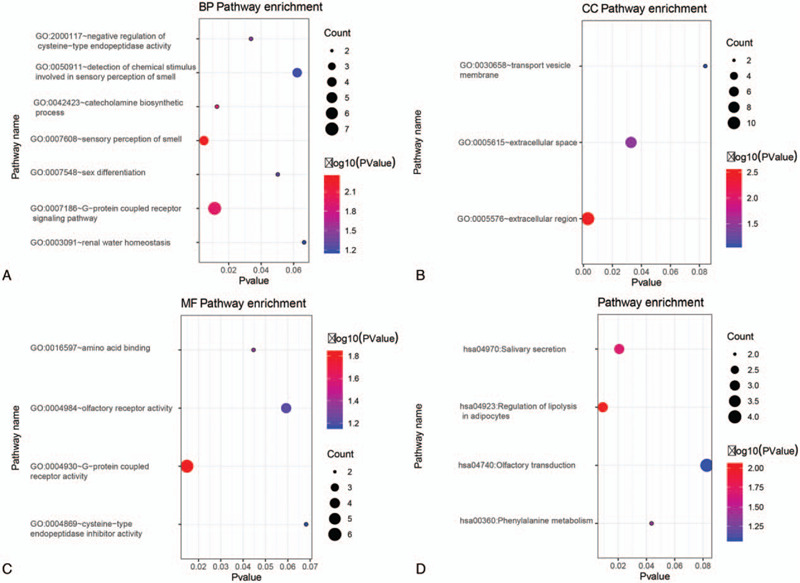
Enrichment analysis of the common differentially expressed genes in high and low Immune/Stromal/ESTIMATE score subgroups. Functional enrichment results of the common differentially expressed genes in the biological process (A), cellular component (B), molecular function (C), and KEGG pathways (D). Count means the number of DEGs enriched in each biological process/pathway. Log10 (Pvalue) means log10 transformed *P*-value.

To further elucidate the functions of these common DEGs in immune/stromal/ESTIMATE subgroups, we performed GO and KEGG enrichment analysis. As shown in Figure 3, these common DEGs were mainly enriched in the protein-coupled receptor signaling pathway, extracellular region, G-protein coupled receptor activity, salivary secretion, and regulation of lipolysis in adipocytes.

### 3.4 Correlation of expression of individual DEGs in overall survival

**Figure 4:**
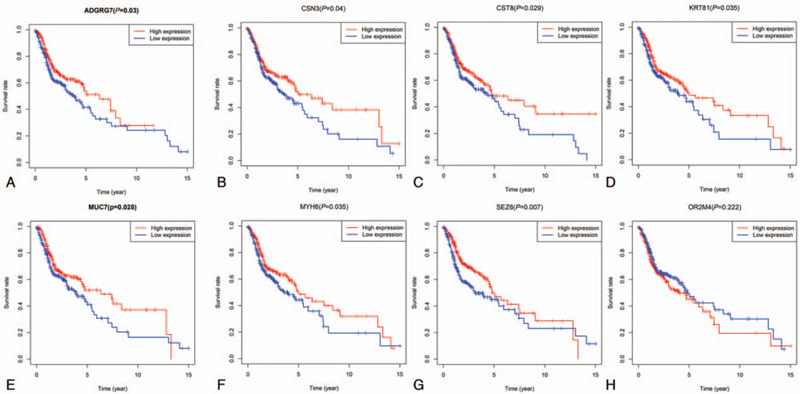
Correlation of the common differentially expressed genes with patient survival. High ADGRG7 (A), CSN3 (B), CST8 (C), KRT81 (D), MUC7 (E), MYH6 (F), and SEZ6 (G) expression is predictive of better survival. Other differentially expressed genes such as OR2M4 (H) were not correlated with patient survival.

To explore the potential roles of the common individual DEGs in overall survival, we conducted Kaplan–Meier survival curves and compared them with the log-rank test. Among the 44 common DEGs, we found 7 genes were closely correlated with patient survival (Fig. 4). They were: ADGRG7 (*P* = .03), CSN3 (*P* = .04), CST8 (*P* = .029), KRT81 (*P* = .035), MUC7 (*P* = .028), MYH6 (*P* = .035), and SEZ6 (*P* = .007). The expression profile of other common DEGs was not significantly related to patient survival.

### 3.5 Protein-Protein interactions of the DEGs

**Figure 5:**
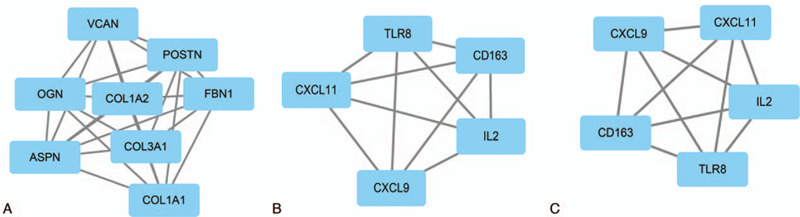
The largest connected models of the differentially expressed genes in high and low Immune/Stromal/ESTIMATE score subgroups. The largest connected models of the differentially expressed genes in high and low Immune (A), Stromal (B), and ESTIMATE (C) score subgroups.

To better understand the interplay among the identified DEGs, we performed PPI network analysis with the online tool of STRING. The maximum connected modules for immune/stromal/ESTIMATE score related DEGs were shown in Figure 5. For immune score related DEG modules, VCAN, POSTN, FBN1, COL1A2, COL3A1, COL1AA1, OGN, and ASPN formed the largest connected module (Fig. 5A). For stromal score related largest DEG module, the connected 5 genes were: TLR8, CD163, IL-2, CXCL9, and CXCL11 (Fig. 5B). For ESTIMATE score related largest DEG module, the connected 5 genes were: CXCL9, CXCL11, IL-2, TLR-8, and CD163 (Fig. 5C). All the connected DEGs were summarized in Table 2. As the table showed, POSTN and OGN were common in all immune, stromal, and ESTIMATE score related DEGs.

**Table 2:** Tumor immunity related key genes in HNSCC.

Group	Hub genes
Immune_score	VCAN, **POSTN, OGN,** COL1A2, ASPN, COL3A1, FBN1, COL1A1,COL10A1, COL8A1, COL6A3, KRT25, KRT73, KRT81
Stromal_score	TLR8, CXCL11, CXCL9, CD163, IL2, ADH1C, GSTA1, UGT1A8, CYP1A1, OMD, **POSTN, OGN,** MMP8, CHIT1, OLFM4, SHH, NKX2-1, OLIG1
ESTIMATE_score	CXCL9, CXCL11, IL2, CD163, TLR8, UGT1A8, CYP1A1, GSTA1, ADH1C, **POSTN, OGN,** OMD, MMP8, CHIT, OLFM4, SHH, OLIG1, NKX2-1

The commonly expressed key genes were empathized in bold.

## 4 Discussion

In the current work, we attempt to identify tumor microenvironment related genes that contribute to HNSCC overall survival in the TCGA database. In particular, by comparing the DEGs between high and low immune/stromal/ESTIMATE score subgroups, we identified 44 common DEGs, of which 7 were significantly related to patient survival. Functional prediction of these common DEGs showed that they were mainly related to extracellular signal transduction and salivary secretion. In PPI analysis, we identified POSTN and OGN were involved in all immune/stromal/ESTIMATE related PPI networks.

The interplay of the tumor and its surrounding microenvironment critically affects tumor evolution, which subsequently impacts subtype classification, recurrence, drug resistance, and the overall prognosis of patients. Tumor purity was found to be closely related to tumor biology and patient survival.^[22]^ Algorithms have been developed and applied to predict tumor purity and screen molecules with prognostic values.^[15,16,23]^ In this study, the immune/stromal/ESTIMATE scores of HNSCC were obtained from the website of the MD Anderson Cancer Center, which was calculated based on the ESTIMATE algorithm.^[16]^ Forty four HNSCC microenvironment related DEGs were identified in our analysis, and 7 of which were verified to be patient survival-related (Fig. 4). They were ADGRG7 (*P* = .03), CSN3 (*P* = .04), CST8 (*P* = .029), KRT81 (*P* = .035), MUC7 (*P* = .028), MYH6 (*P* = .035), and SEZ6 (*P* = .007).

Of these 7 survival-related genes, CSN3, KRT81, MUC7, and MYH6 has been elucidated to take part in tumor progression. Functional analysis of ADGRG7, CST8, and SEZ6 in tumor biology has not been reported. CSN3 gene encodes the protein of CSN, which acts as a protein kinase and a demethylase in mammalian cells, and is essential for the maintenance of cell proliferation in the mouse embryonic epiblast and associated with the tumorigenesis process in osteosarcoma and hepatocellular carcinoma.^[24]^ KRT81 was found to be related to progression-free survival in Esophageal Adenocarcinoma patients^[25]^ and the overall survival in early-stage non-small cell lung cancer.^[26]^ MUC7 expression is an independent predictor of adverse clinical outcomes in patients with clear cell renal cell carcinoma.^[27]^ Furthermore, MUC7 was found as an independent risk factor for the recurrence of bladder cancer with muscle invasion.^[28]^ MYH6 has been found to play an important role in tobacco independent disease development and progression, including HNSCC,^[29]^ and was deemed as a novel putative cancer gene.^[30]^ These results give further evidence to our results that these tumor microenvironment related genes affect tumor progression and patient survival. SEZ6 is important for the development of neuronal dendrites and synapses and takes part in the development of chronic hyperalgesia and neuroinflammation after nerve injury.^[31]^ However, the involvement of SEZ6 in tumor initiation and progression has not been reported. The role of ADGRG7 and CST8 in tumor biology has not been reported. Our results propose the potential of these genes for targeted therapy and give the rationale of focusing on these genes for further studies.

Protein-protein interaction refers to the physical binding of 2 or more proteins as responses to different disturbances and circumstances, which provide considerable adaptability for biological cells to adapt flexibly to the changing environmental conditions.^[32]^ In this study, we identified the DEGs between high and low immune/stromal/ESTIMATE score subgroups, and the interaction of these DEGs was calculated and visualized by STRING and the MCODE plug-in in Cytoscape. The connected nodes were selected and summarized in Table 1. These connected genes may represent the main functional changes in tumor progressionand functional analysis of these connected genes may provide potential targets for gene therapy. The respective top module for immune/stromal/ESTIMATE classification was demonstrated in Figure 5. Interestingly, we found POSTN and OGN were common in all classification, indicating their key role in the tumor microenvironment and tumor progression. The function of these 2 genes has been fully elucidated. POSTN has been reported to participate in the epithelial-to-mesenchymal transition, radio-resistance, angiogenesis, and invasion of HNSCC.^[33–36]^ Furthermore, a recent study revealed that fibroblasts secreted POSTN promotes cancer stemness in HNSCC by activating protein tyrosine kinase 7.^[37]^ This result was consistent with our analysis, indicating the key role of POSTN in mediating the cross-talk of tumor cells and tumor microenvironment, and in modulating tumor cell biology. Similarly, OGN has also been identified as a key regulator in tumor proliferation, invasion, and epithelial to mesenchymal transition in colorectal, breast, and ovarian cancers.^[38–40]^ Recently, OGN was reported to enhance T lymphocyte infiltration in colorectal cancer,^[41]^ indicating its role in regulating tumor microenvironment. This result is supportive of our bioinformatics analysis. In another bioinformatic analysis for screening the potential diagnostic and therapeutic targets for laryngeal carcinoma, OGN was also identified as a hub gene.^[42]^ The precise mode of action for ONG in the cross-talk between HNSCC and tumor microenvironment still needs further elucidation.

## 5 Limitations

There are limitations to this study. First, only data from the TCGA project was included, no external validation was performed. Second, only bioinformatic analysis was conducted in this study, molecular experiments were needed to further validate the role of selected genes in this study. Third, some well-documented risk factors for HNSCC, such as HPV infection, chemotherapy, and radiotherapy were not considered in this study, which makes some inevitable noise to the result.

## 6 Conclusions

Despite limitations, conclusions still could be drawn from this study. In this study, we screened a list of tumor microenvironment related genes for tumor progression and patient survival in HNSCC. These genes have the potential of diagnostic, predicting, and therapeutic values for HNSCC and thorough research on these genes may broaden the understanding of the potential relationship between tumor microenvironment and HNSCC.

## Author contributions

**Conceptualization:** Di Meng.

**Data curation:** Di Meng, Tongjun Liu.

**Formal analysis:** Tongjun Liu.

**Methodology:** Feng Ma.

**Supervision:** Mingguo Wang.

**Writing – original draft:** Di Meng.

**Writing – review & editing:** Di Meng, Mingguo Wang.

## Abbreviations

Abbreviations: BP = biological processes, CC = cellular components, DAVID = The Database for Annotation, Visualization, and Integrated Discovery, DEGs = differentially expressed genes, ESTIMATE = Estimation of Stromal and Immune cells in Malignant Tumor tissues using Expression data, FDR = false discovery rate, GO = gene ontology, HNSCC = Head and neck squamous cell carcinoma, KEGG = Kyoto Encyclopedia of Genes and Genomes, MCODE = Molecular Complex Detection, MF = molecular functions, PPI = protein-Protein-Interaction, TCGA = the cancer genome atlas.
